# *i*AK692: A genome-scale *metabolic model of Spirulina platensis C1*

**DOI:** 10.1186/1752-0509-6-71

**Published:** 2012-06-15

**Authors:** Amornpan Klanchui, Chiraphan Khannapho, Atchara Phodee, Supapon Cheevadhanarak, Asawin Meechai

**Affiliations:** 1 Systems Biology and Bioinformatics Research Group, Biochemical and Pilot Plant Research and Development Unit, King Mongkut’s University of Technology Thonburi, National Center for Genetic Engineering and Biotechnology, Bangkok, Thailand; 2 Department of Chemical Engineering, Faculty of Engineering, King Mongkut’s University of Technology Thonburi, Bangkok, Thailand; 3 Devision of Biotechnology, School of Bioresources and Technology, King Mongkut’s University of Technology Thonburi, Bangkok, Thailand

**Keywords:** *Spirulina platensis* C1, Cyanobacteria, Metabolic network reconstruction, Genome-scale metabolic model, Flux balance analysis

## Abstract

**Background:**

*Spirulina (Arthrospira) platensis* is a well-known filamentous cyanobacterium used in the production of many industrial products, including high value compounds, healthy food supplements, animal feeds, pharmaceuticals and cosmetics, for example. It has been increasingly studied around the world for scientific purposes, especially for its genome, biology, physiology, and also for the analysis of its small-scale metabolic network. However, the overall description of the metabolic and biotechnological capabilities of *S. platensis* requires the development of a whole cellular metabolism model. Recently, the *S. platensis* C1 (*Arthrospira* sp. PCC9438) genome sequence has become available, allowing systems-level studies of this commercial cyanobacterium.

**Results:**

In this work, we present the genome-scale metabolic network analysis of *S. platensis* C1, *i*AK692, its topological properties, and its metabolic capabilities and functions. The network was reconstructed from the *S. platensis* C1 annotated genomic sequence using Pathway Tools software to generate a preliminary network. Then, manual curation was performed based on a collective knowledge base and a combination of genomic, biochemical, and physiological information. The genome-scale metabolic model consists of 692 genes, 837 metabolites, and 875 reactions. We validated *i*AK692 by conducting fermentation experiments and simulating the model under autotrophic, heterotrophic, and mixotrophic growth conditions using COBRA toolbox. The model predictions under these growth conditions were consistent with the experimental results. The *i*AK692 model was further used to predict the unique active reactions and essential genes for each growth condition. Additionally, the metabolic states of *i*AK692 during autotrophic and mixotrophic growths were described by phenotypic phase plane (PhPP) analysis.

**Conclusions:**

This study proposes the first genome-scale model of *S. platensis* C1, *i*AK692, which is a predictive metabolic platform for a global understanding of physiological behaviors and metabolic engineering. This platform could accelerate the integrative analysis of various “-omics” data, leading to strain improvement towards a diverse range of desired industrial products from *Spirulina*.

## Background

*Spirulina* (*Arthrospira) platensis* is a filamentous non-N_2_-fixing cyanobacterium that has become important as a source for commercially produced nutraceutical compounds, as this cyanobacterium utilizes sunlight and CO_2_ to produce chemical compounds that are essential for life. *Spirulina* has been consumed as a protein source for many years by North Africans and Mexicans [1] because it contains high amounts of healthy nutritional molecules such as beta-carotene, phycocyanin, vitamins, trace minerals, and polyunsaturated fatty acids [2]. Recently this cyanobacterium has played an important role in a wide range of applications in the nutraceutical industry, including human food supplements and animal feed [3]. Moreover, many scientific articles have reported the therapeutic benefits of this microorganism, such as helping to prevent heart disease, cancer, and diabetes [4]. Furthermore, *S. platensis* is potentially one of the algae capable of producing bioenergy and renewable energy, which could help to decrease the effects of global-warming [5]. Among the diverse range of cyanobacterial species, *S. platensis* is capable of growing in outdoor environments at a high rate [6]. In terms of cellular capacities, many of its bioactive compounds could be inexpensively produced by photosynthesis. These facts plus its nutritional value make *S. platensis* an attractive photobiological cell factory.

The growing availability of genomic sequences and software technologies has made it possible to reconstruct genome-scale metabolic networks of various organisms. Genome-scale metabolic models come from the systematic reconstruction of all cellular biochemical reactions according to the genetic information of a given organism [7]. A vast number of applications of a reconstructed metabolic network have been reported and include such possibilities as genome annotation and metabolic engineering [7]. Knowledge of the presence or absence of specific pathways in a given organism can help to improve the quality of genome annotation [8]. Furthermore, after the metabolic pathways are initiated, this reconstructed metabolic network becomes a useful tool for applications in the area of metabolic engineering, the general goal of which is to redistribute fluxes within a metabolic network towards a desired goal [9]. Reconstruction of the metabolic network is also necessary for *in silico* predictions of gene functions and the metabolic capabilities of an organism [10]. By applying flux balance analysis (FBA) technique [11,12], the metabolic network may be converted to a genome-scale model, allowing a qualitative assessment of the relationship between genotypic and phenotypic behaviors, and a global estimation of flux distributions within the metabolism of an organism, which cannot possibly be measured using a standard experimental design. Currently, one popular tool for investigating complex stoichiometric metabolic models is the constraint-based reconstruction and analysis (COBRA) toolbox [13,14] with MATLAB. This technique relies on linear programming (LP) and a given set of various appropriate constraint parameters known from experiments. Numerous successes have been reported using these methods as the tools to elucidate *in silico* models (virtual organisms) [15-17].

Various genome-scale metabolic models of many organisms are currently available [18]. However, of cyanobacterium, only *Synechocystis* sp. PCC6803 has been developed by independent research teams around the world [19-21]. Each proposed model provides informative knowledge on rational bioenergy production by *Synechocystis* sp. PCC6803 as a photobiological cell factory. With such an impressive advantage of S*. platensis,* especially as a nutraceutical, as previously mentioned, S*. platensis* has become one of the preferred choices for a sustainable photobiological cell factory. Unfortunately, there have only been a limited number of attempts to computationally analyze the metabolism of *Spirulina*. A simple metabolic flux model of *S. platensis* consisting of 22 reactions was proposed by Meechai *et al*[22]. This model was used to predict rate limiting enzymes for the production of gamma-linolenic acid. A larger metabolic network of *S. platensis* comprising 121 reactions and 134 metabolites was formulated by Cogne and his team [23]. This model accounted for central metabolic pathways, anaplerotic reactions, energy metabolism reactions, anabolic reactions, synthesis of macromolecules, biomass and growth-associated exopolysaccharides (EPS). However, these two models did not provide the whole-cell characteristics and metabolic capabilities of *S. platensis*. Recently, the genome sequence of *S. platensis* C1 became available [24], together with an increasing number of studies of its physiological and molecular levels. These data have enabled a genome-scale metabolic model reconstruction of *S. platensis*.

This paper presents the first genome-scale metabolic model of *S. platensis* (i.e., *i*AK692), representing global growth behaviors under three different growth conditions: autotrophic, heterotrophic, and mixotrophic. The metabolic network is based on the *S. platensis* C1 genome, a collective knowledge base, and extensive manual curation. Computational simulation was performed using COBRA toolbox [13,14]. The results from *in silico* predictions were further validated with experimental evidence. Various analyses of the *i*AK692 model were performed to identify active reactions and essential genes under each growth condition. Moreover, phenotypic phase plane (PhPP) [25] analysis was carried out to predict the metabolic states of *i*AK692 during autotrophic and mixotrophic growths. The *i*AK692 model not only provides further physiological knowledge of the cellular system, but is also a valuable platform for integrating multilevel “-omics” data, which could provide further insight towards increasing the number of desired industrial bioproducts from *Spirulina*.

## Results and discussion

### Metabolic model reconstruction

#### Reconstruction of a draft model

**Figure 1:**
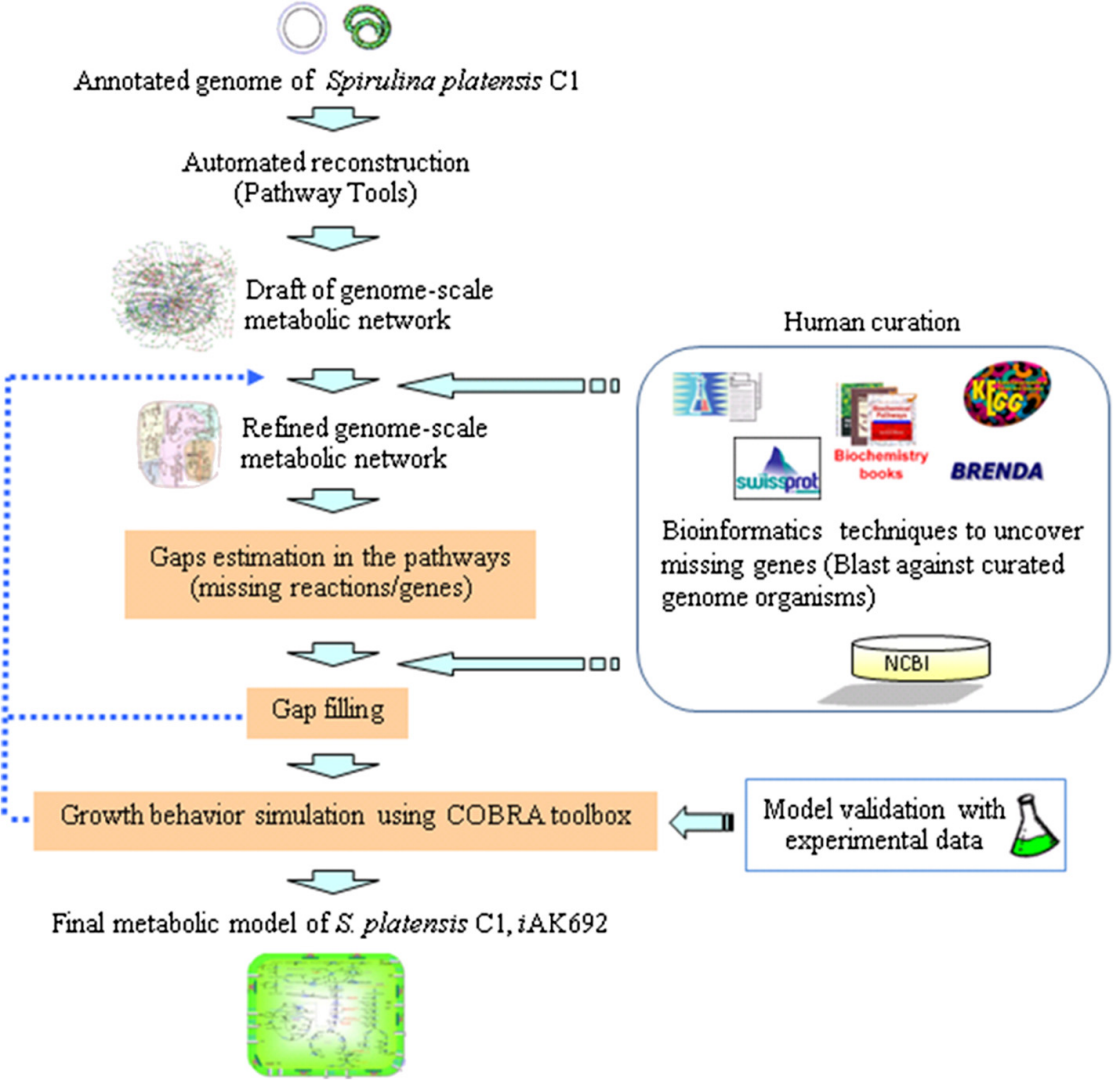
**The iterative procedure used to reconstruct the genome-scale metabolic model of*S. platensis*C1.** The draft of the metabolic network was automatically established using Pathway Tools software. The manual curation was performed based on a combination of *Spirulina*-related journal publications and biochemical books and databases. Missing reactions in the pathway were manually estimated and filled. Blast program was used in order to find a potential corresponding gene. The accepted network was further studied via a structural analysis and simulation based on the COBRA toolbox [13,14.]

In order to build a genome-scale metabolic model of *S. platensis* C1, the genome-scale metabolic network was reconstructed according to a series of extensive refinements (Fig. 1 and see also Materials and Methods). The genome of *S. platensis* C1 is deposited in the GenBank Database (NCBI ID 67617) [24]. The estimated genome size for the 63 DNA scaffolds is approximately 5.9 Mb. A total of 6,176 open reading frames (ORFs) were predicted; 3,759 ORFs (61 %) were well annotated with gene functions; and 2,372 ORFs (38 %) were identified as putative or hypothetical proteins (Table 1). Reconstruction of the model was initiated with automated construction using Pathway Tools software [26,27]. Using this fully computational procedure, a draft metabolic network, called the “Draft model”, was generated based on the annotated genome data. This automated process facilitates the top-down reconstruction approach, giving an overview network and visualization; therefore, it provides the maximum possible number of pathways, each of which has different numbers of reactions and genes.

**Table 1:** Characteristics of model size after each reconstruction step

**Features**	**Numbers**		
**Genome information**			
Genome size (bp)	5,934,248		
Open reading frames(ORFs)	6,176		
Annotated genes	3,759		
**Reconstruction metabolic model**	**Draft model**	**Refined model**	**Final model**
**Genes**			
ORFs	575	692	692
% ORF of genome	9.3	11.2	11.2
**Reactions**	1,661	688	875
Internal enzymatic reaction	1,661	688	699
Gene-associated	849	650	650 (93 %)
No gene association	812	38	49 (7 %)
Exchange reaction	0	0	176
**Metabolites**	1542	658	837

The Pathway Tools software [26,27] predicts the metabolic network for *S. platensis* C1 based on the gene name or EC number matching method. This preliminary model accounted for 102 pathway frames (including a number of pathway variants), 557 genes corresponding to 1,661 biochemical reactions, and 1,542 metabolites. A summary of the draft model is shown in Table 1. The pathways were also predicted and a large number of the pathway reactions were obtained. We also found that one gene could participate in more than one reaction of different pathways. Thus, this process built 102 candidate pathways and 812 out of 1,661 reactions were presented and showed no gene association. However, the quality of the predicted large-scale reconstructed network depends on the quality of the initial annotated data. Furthermore, the reconstructed network must express the biology and physiology of *S. platensis* C1; for instance, whether or not the predicted pathways or reactions present in *S. platensis* C1 and the numbers of incomplete pathways (missing gene and reaction) indicate a disconnection in the network. Hence, manual-intensive curation was subsequently performed to increase the accuracy of the draft auto-generated network.

### Metabolic network refinement

Biochemical information about cyanobacteria was obtained from the literature and biochemistry textbooks, and online biochemical databases were used to research several iterative methods of reconstruction and were used to refine the draft model; this is referred to as the “Refined model”. In more detail, the predicted pathways/reactions that have not been reported for *Spirulina* physiology, such as cholesterol biosynthesis I, II, and III pathways, were eliminated from the network. Pathway Tools mapped only one gene, *crtB*, encoding phytoene synthase, onto two reactions out of twenty-two reactions in these pathways for the draft model. However, we found that the phytoene synthase enzyme was involved in the presence of the carotenoid biosynthesis pathway. Since carotenoid is an important molecule in photosynthesis, the synthesis pathway of this compound was retained.

Then the Blast algorithm [28,29] and the tool for the protein domain prediction against pfam databases [30] were used to determine the enzymatic gene functions needed to complete the pathways where no gene could be found in the automated metabolic reconstruction. A total of 135 genes were annotated and added to the network. Missing reactions (referred to as gaps) that resulted in dead-end metabolites and prevented the computational simulation of cell growth were identified and filled in. This procedure was continued until all of the biomass components in Table 2 were included. After manually updating the gap filling process, we also attempted to search for the genes in the genome, which can be associated with the added reactions. A total of 35 reactions from closely related organisms were added to complete the connectivity of the network. The refined model contains 692 metabolic genes, 658 metabolites, and 688 biochemical reactions (Table 1). The number of many-to-many relationships between reactions and genes presented in the network is 207 reactions.

**Table 2:** ***i*AK692 biomass composition equation**

**Component**	**Molar ratio**	**Component**	**Molar ratio**
Carbohydrate (16 %)*	0.1230		
CMP-*N*-acetylneuraminate	0.0220	Glycogen	0.0430
Cyclitol	0.0510	Peptidoglycan	0.0300
dTDP-rhamnose	0.7320	UDP-D-glucose	0.1240
Protein (68 %)*	0.8404		
Alanine	0.1010	Lysine	0.0360
Arginine	0.0530	Methionine	0.0180
Aspartate	0.0980	Phenylalanine	0.0390
Cysteine	0.0070	Proline	0.0380
Glutamate	0.1320	Serine	0.0540
Glycine	0.0860	Threonine	0.0540
Histidine	0.0140	Tryptophan	0.0090
Isoleucine	0.0580	Tyrosine	0.0330
Leucine	0.0940	Valine	0.0760
Lipid (11 %)*	0.0182		
DGDG	0.1310	SQDG	0.1470
MGDG	0.3200	Triglyceride	0.1860
Glycerol	0.2160		
DNA (0.88 %)*	0.0039		
dATP	0.2790	dGTP	0.2220
dCTP	0.2220	dTTP	0.2790
RNA (3.12 %)*	0.0130		
ATP	0.2620	GTP	0.3220
CTP	0.2000	UTP	0.2160
Antenna chromophore (1 %)*	0.0016		
Cholorophyll a	0.0016		

* % total dry weight of major cellular composition estimated from *S. platensis* cell grown under autotrophy [23]. The stoichiometric coefficient of each building block was retrieved and expressed on the basis of one mole of dry cell. DGDG: digalactosyldiacylglycerol; MGDG: monogalactosyldiacylglycerol; SQDG: sulfoquinovosyldiacylglycerol.

#### Generation of the computational genome-scale model (iAK692)

In order to make the computation-ready model, referred to as the “Final model”, the balance between each element, including metabolites and protons (H^+^), reaction directionality, transport reaction, and the completely characterized biomass equation were curated and added to the refined model. We checked the balance of protons in a reaction and also found that different databases contain some contradictory information. In this reconstruction, we used Metacyc [31] as the key reference for H^+^ balancing. Additionally, the stoichiometry and reversibility of the reactions were manually verified and assisted by biochemical reaction databases such as KEGG [32] and Brenda [33]. We also considered the direction of the reaction on the basis of thermodynamics. If the reaction consumed high-energy compounds such as ATP, the reaction was designated irreversible. Considering only the internal reactions of the model, there are 558 irreversible and 317 reversible reactions. The cofactors, i.e, NADPH, and NADH involved in the reactions were manually curated based on information from literature [7] and the published genome-scale model of *Synchocystis* sp. PCC6803 [19]. The system boundary was defined across the cytoplasmic membrane and environment in terms of transport reactions. Diffusion was assumed to be the mechanism for transporting nutrients between cytoplasm and extracellular environment. Thus, transporter genes were not assigned in this first large-scale model of *S. platensis*. Instead, a total of 88 exchange reactions were included in the model to ensure that organic nutrient metabolites (i.e., amino acid and sugar), inorganic nutrient metabolites (i.e., phosphate, sulfate, nitrate, and potassium), gaseous metabolites (i.e. O_2_ and CO_2_), photons, and water could enter and leave the system in response to the physiological state. We are aware that the transport mechanisms across the cell membrane should be considered in this study because of their possible effect on the model. This is an issue that we will address in a future study.

In order to determine the capability of a genome-scale model, the most popularly used objective function in FBA is normally the biomass objective function (BOF). A biomass equation represents all necessary precursors that form the cellular biomass. Thus, 14 reactions with no gene association were added for the synthesis of intermediate macromolecules, i.e. proteins, carbohydrates, lipids, RNA, DNA, and chlorophyll, which are considered to form the major composition of biomass. The biomass formation equations were obtained based on data from different sources of experimental efforts for *S. platensis* under autotrophic conditions [23] (Table 2). Moreover, the net reaction for all light-dependent reactions was obtained from a previous report [34]. All reactions in the reconstructed metabolic network are listed and shown in Additional file 1: Table S1.

**Figure 2:**
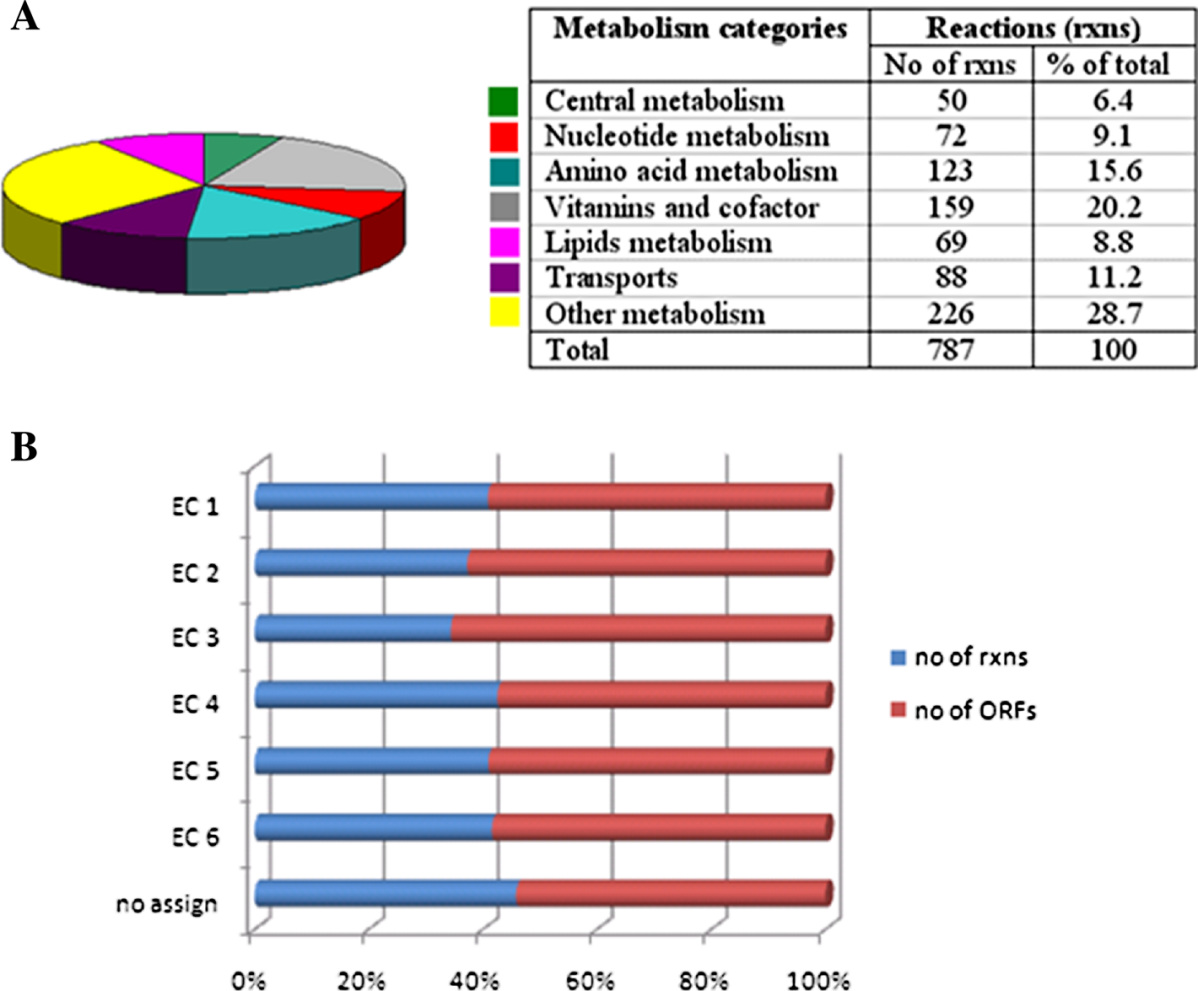
**Classification of metabolic reactions in the*S. platensis*C1 model. **(A) Distribution of each metabolic reaction; the table shows the numbers for the total reaction. (B) Percentage of reactions and genes classified based on the EC number. EC 1: oxidoreductases; EC 2: transferases; EC 3: hydrolases; EC 4: lyases; EC 5: isomerases; EC 6: ligases.

The different methods of reconstruction resulted in the final model, *i*AK692, and account for 692 metabolic genes, 837 metabolites, 176 exchange reactions, and 699 internal biochemical reactions, including 650 gene-associated reactions and 49 with no gene association (Table 1). A total of 875 reactions included in the model were designated as belonging to the high level of metabolism (Fig. 2A). The functional classification of the ORFs included in the reconstruction is summarized in Figure 2B, based on the Enzyme Commission (EC) number [35]. The majority of ORFs are associated with the transferase enzyme (EC 2).

#### Network topology of iAK692

The structural organization of *i*AK692 was characterized by an analysis of metabolic connectivity via reactions within the network. Most of the metabolites in the network have few connections, whereas a few metabolites participate in a large number of reactions. The top 10 most highly connected metabolites are ATP, ADP, phosphate, diphosphate, NADP, NADPH, CO_2_, NAD, NADH, and O_2_. These frequently used metabolites were found to be involved in energy metabolism, such as ATP and ADP, and in redox metabolism, such as NADPH and NADP (Table 3). These metabolic hubs serve as key compounds related to the core metabolism of the organism in transferring specific biochemical groups such as phosphate groups, redox equivalents, amino groups and acetyl groups. In addition, connectivity was compared to other genome-scale models, including *Synechocystis* sp. PCC6803 (*i*Syn669) [20], *Escherichia coli (i*AF1260*)*[36], and yeast (*i*FF708) [37]. It was found that *i*AK692 has a similar network topology and metabolite hub to these models (Table 3). It should also be noted that these metabolites are involved in energy and redox metabolism, representing the currency of biological life. The topology also indicates that in the organization of the network a few hubs dominate the overall connectivity of the network and the network eventually disintegrates into isolated clusters. These characters show to what degree different components of the cellular metabolism are interconnected. Perturbations in cellular behavior, such as changing a few fluxes in metabolism, can affect the entire metabolism. Hence, studying the structural organization of the network provides hints for discovering corresponding regulatory mechanisms of the cell.

**Table 3:** **Numbers of metabolite connectivity of top 10 metabolites in the*i*AK692 metabolic network compared to those in other published models**

**Metabolite**	**Connectivity**			
	***i*AK692**	***i*Syn669**	**E.coli**	**yeast**
ATP	134	144	338	166
ADP	92	103	253	131
PI	92	108	81	113
PPI	84	97	28	-
NADP+	84	64	39	61
NADPH	83	63	66	57
CO_2_	72	72	53	66
NAD+	70	46	79	58
NADH	69	42	75	52
O_2_	57	36	40	31

#### Validation of iAK692

*S. platensis* C1 naturally grows under autotrophic conditions using carbon dioxide as a carbon source and converting light as cellular energy, like other cyanobacteria. Several strains of these microalgae, including *S. platensis*, were recently researched for their potential to grow under heterotrophic and mixotrophic environments [38]. However, there have been no reports of either mode of cultivation in the *S. platensis* strain C1. Thus, we investigated the cellular properties of heterotrophic and mixotrophic growth in order to gain more basic physiological knowledge of *S. platensis* C1. Subsequently, we validated the reconstructed metabolic network by comparing the predicted results from *in silico* simulations with the experimental results. The measured maximum specific growth rates of *S. platensis* C1 under the three growth conditions are summarized in Table 4. More details of the results are shown in Additional file 2: Table S2.

**Table 4:** **Constraints for metabolite uptake rates used for model simulation, and the comparison of the predicted growth rate by the*in silico*model and observed growth rate from the experiments in each growth condition**

**Growth conditions**	**Constraints of consumed metabolites (mmol/mmol dry cell/h)**					**Maximal specific growth rate (1/h)**	
	Bicarbonate	Phosphate	Nitrate*	Sulfate*	Glucose**	*In silico*	*In vivo*
Autotrophic	0.20	0-0.0056	0-0.040	0-0.0014	0	0.0257	0.0255
Heterotrophic	0	0-0.0056	0-0.040	0-0.0014	0.017	0	0
Mixotrophic	0.20	0-0.0056	0-0.040	0-0.0014	0.017	0.0334	0.0262

* data from ref. 38.

** data from ref. 39.

The basic capabilities of *i*AK692 were evaluated based on constraint-based modeling using the FBA technique [11,12] to quantitatively predict growth under the three metabolic modes. Assuming a steady state, FBA simulates the mass balance of all metabolites derived from the stoichiometric reactions together with constraints and the objective function. In this study, we set the biomass flux as the objective function.

For autotrophic growth, cells synthesize organic molecules for biomass formation from inorganic compounds and sunlight. The maximum specific growth rate of *S. platensis* C1 was demonstrated in batch cultivation (Table 4). The uptake rates of the main metabolites, bicarbonate and phosphate, were measured and used as the constraints of the simulation. In order to assess the predictive potential of the model, we simulated the *in silico* model based on minimal media consumption, as shown in Table 4. The constraints of certain metabolite uptake rates were obtained from the literature [38,39]. The photon flux was set to be between zero and 100 μEinstein/m^2^/s with a fixed uptake rate of 0.20 mmol/mmol dry cell/h of bicarbonate. The results show that the maximum *in silico*-specific growth rate was similar to the maximum specific growth rate measured from the experiments. The experimentally determined value and the computationally predicted value were 0.0255 and 0.0257 1/h, respectively. On the other hand, when the photon flux was omitted (i.e., set to 0 μEinstein/m^2^/s), the *in silico* cell could not grow (i.e., the maximum specific growth rate was 0 1/h. This simulation results show a consistency with the control experiment, in which no growth was found.

For the heterotrophic mode, *S. platensis* C1 was cultivated on glucose in the dark. The results show that the cell failed to grow heterotrophically (Table 4). In the simulation, we set the glucose uptake rate equal to 0.017 mmol/mmol dry cell/h with no light. The predicted value of growth was zero (Table 4), showing an agreement between *in vivo* and *in silico* conditions. Considering the genomic data, *S. platensis* C1 has the *hex* gene encoding the hexokinase enzyme, which converts glucose molecules into glucose-6-phosphate. This is the first important step in the glycolysis pathway, which allows cells to metabolize glucose as a carbon source. However, a lack of the ability to utilize sugar in the dark was recently reported in some strains of *Spirulina*[40]. Therefore, this agreement in the results may stem from no or a loss of function of the gene product when growing for a long period of time under autotrophic conditions in the laboratory. Moreover, the glucose concentration had no effect on the growth of *S. platensis* under the heterotrophic conditions reported in a previous study [41].

In the mixotrophic mode with carbon dioxide and glucose as carbon sources and light as the energy source, the measured growth rate under this condition was found to be slightly higher than that of the autotrophic mode (Table 4). The model simulation under mixotrophic conditions also showed a higher growth rate (0.0334 1/h), although to a different extent. Interestingly, this growth rate corresponds to that of an increase in biomass formation during the mixotrophic cultivation of another *Spirulina* strain by Lodi and his team [39]. They suggested that the mixotrophic culture had the highest growth rate because the heterotrophic and autotrophic metabolism processes might be active in parallel. However*,* the current experimental study for *S. platensis* C1 has showed that the mixotrophic growth rate (0.0262 1/h) is only slightly higher than the autotrophic growth rate (0.0255 1/h) (Table 4). We also modeled the *hex* gene knockout that results in zero flux of this reaction. The results show that the growth rate decreased from 0.0334 1/h to 0.0257 1/h (data not shown). Therefore, the different growth behaviors found for the different physiological properties and *in silico* predictions might require further experimental verification in order to discover further explanations and make new discoveries. All profiles of the flux distributions of the three growth conditions are presented in Additional file 2: Table S2.

#### Reaction activity and flux variability analyses

**Figure 3:**
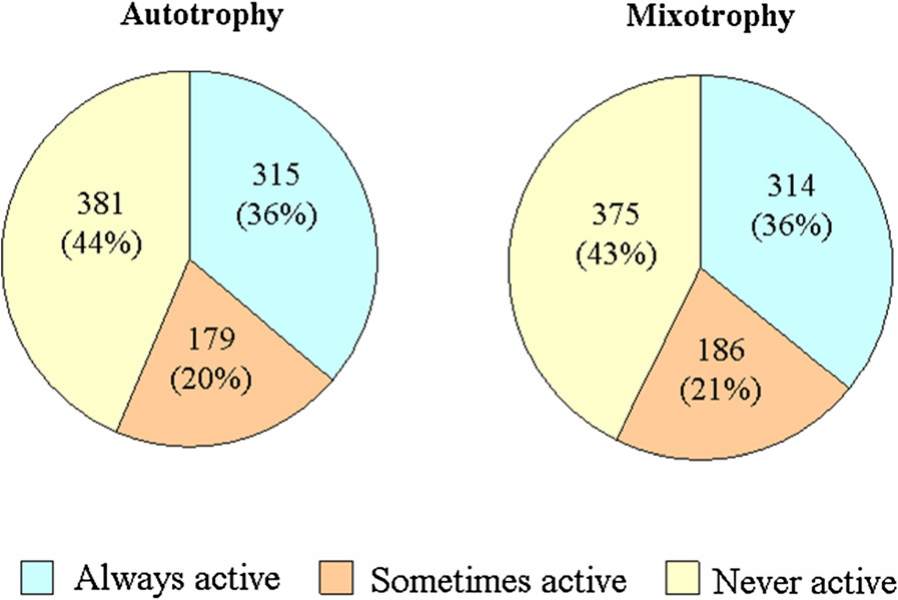
**Comparison of predicted active reactions under different growth conditions.** (A) autotrophy, (B) mixotrophy.

The result validation demonstrates that the *in vivo* growth rates of *i*AK692 are consistent with the experimental growth rates found under the three different modes. In order to investigate the flux distributions in terms of active reactions, this model was only used for the simulation of autotrophic and mixotrophic growths because the cells failed to grow under heterotrophic conditions. The flux results show that *i*AK692 had 322 (41 %) and 307 (39 %) active reactions under the autotrophic and mixotrophic conditions, respectively. These two numbers of active reactions are close to those found in previous studies of other genome-scale models such as those for *Clostridium beijerinckii*[42], *E. coliStaphylococcus aureusHelicobacter pylori* and yeast [43], which comprise around 300 active reactions. For a more precise determination of active reactions in each growth condition, we performed flux variability analysis (FVA) [44] to determine possible ranges of fluxes of all reactions that still satisfy the same optimal growth. Here, we classify the reaction activity into 3 categories based on the FVA results: (i) a reaction is considered “always active” if min/max flux values are non-zero with the same sign, (ii) a reaction is considered “sometimes active” if the range of possible fluxes contains zeroes, and (iii) a reaction is considered “never active” if min/max flux values are equal to zero during optimal growth. To satisfy the optimal autotrophic growth, we found that 315 (36 %) reactions are always active; 179 (20 %) reactions are sometimes active, and 381 (44 %) reactions are never active (Fig. 3). On the other hand, to achieve its optimum growth in mixotrophic condition 314 (36 %) reactions are always active; 186 (21 %) are sometimes active, and 375 (43 %) are never active for the *S. platensis* (Fig. 3). It is noted that there is a total of 494 and 500 “active reactions” (both sometimes and always active) for autotrophic and mixotrophic conditions, respectively. It was observed that all the 494 reactions found active under the autotrophic growth are also active under the mixotrophic growth. The six remaining reactions that are found active only under the mixotrophic growth are shown in Table 5. These reactions belong to the pathways involving glucose exchange, glycolysis, arginine and proline metabolism, and cofactor and prosthetic group biosynthesis. We think that these pathways are necessary for the conversion of external glucose to intermediates in the synthesis of macromolecules needed for mixotrophic cell growth. All profiles of FVA of both growth conditions are presented in Additional file 3: Table S3.

**Table 5:** **List of reactions found active only in*i*AK692 grown under mixotrophic condition**

**Reaction ID**	**Reaction name**	**Reactiuon equation**	**Pathway**	**Responsible gene**
SP0001	glucokinase	Alpha-D-glucose + ATP -- > alpha-D-glucose-6-phosphate + ADP + H+	Glycolysis	SPLC1_S271400
SP0111	ornithine acetyltransferase	L-glutamate + acetyl-CoA -- > N-acetyl-L-glutamate + coenzyme-A + H+	Arginine and proline metabolism	SPLC1_S202710
SP0267	glutamate-cysteine ligase	L-cysteine + L-glutamate + ATP -- > L-gamma-glutamylcysteine + phosphate + ADP + H+	Cofactor and prosthetic group biosynthesis	SPLC1_S361150
SP0268	glutathione synthetase	glycine + L-gamma-glutamylcysteine + ATP -- > reduced-glutathione + phosphate + ADP + H+	Cofactor and prosthetic group biosynthesis	SPLC1_S531620
LGLUCtex	Glucose transport	alpha-D-glucose < −− > alpha-D-glucoseXT	D-glucose exchange	
LGLUCtexX	Glucose transport	alpha-D-glucoseXT < −− > alpha-D-glucoseXTX	D-glucose exchange	

### Metabolic gene essentiality analysis

**Figure 4:**
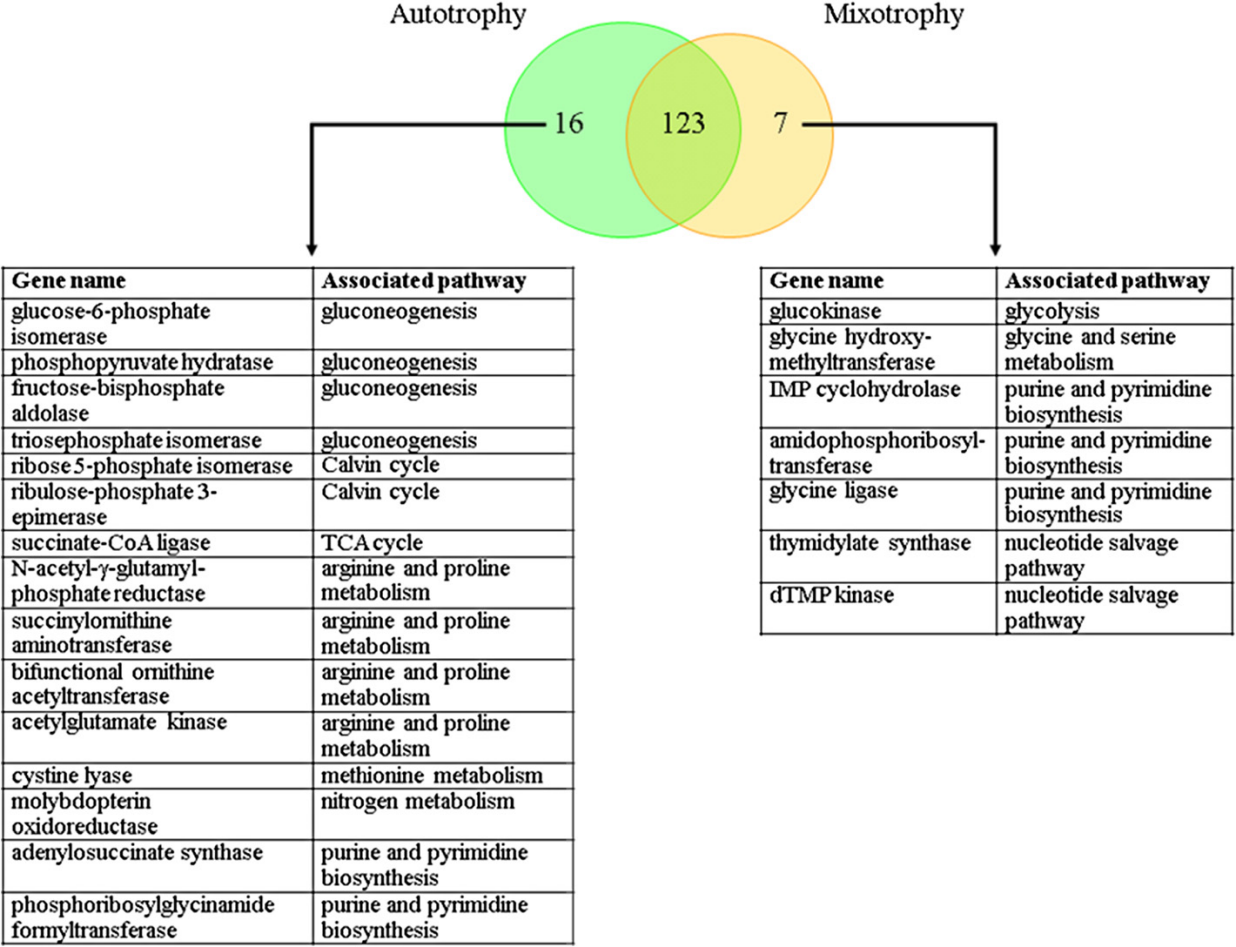
Comparison of essential genes under different growth conditions.

The capacity of *i*AK692 for predicting the growth behavior when it suffers gene deletion was evaluated. Like many other constraint-based models [45], the *i*AK692 model contains a list of gene-protein-reaction interactions indicating which genes are connected with each reaction in the metabolic network. The essentiality of each gene can be determined by constraining its associated reaction not to carry flux. Therefore, the network reaction(s) associated with each gene was deleted, one by one, by setting both the upper and lower bounds of a reaction to zero and optimizing for the biomass formation. In this study, the *i*AK692 model accounting for 875 biochemical reactions and 837 metabolites was employed to identify the essential genes for survival under autotrophic and mixotrophic growth conditions using the MOMA platform [46]. It was found that 139 and 130 genes were essential for the growth of *i*AK692 under autotrophic and mixotrophic conditions, respectively (Fig. 4). A list of these essential genes can be found in the Additional file 4: Table S4. The total number of 130 essential genes for the mixotrophy was about 19 % (130 of 692) of the total genes. This number is close to those found in other published models -- 15 % (187 of 1,260 genes) in *E*. *coli* (*i*AF1260) [36], and 10 % (148 of 708 genes) in yeast (*i*FF708) [37]. It is noted that there were 123 common essential genes between the two growth conditions. These common genes can be considered core metabolic genes for the growth of *i*AK692. Fig. 4 also shows a list of unique essential genes for each growth condition. Not surprisingly, the unique essential genes for autotrophy were the genes associated with the Calvin cycle and gluconeogenesis pathways that are responsible for photosynthesis and glucose formation, respectively.

#### Phenotypic phase plane analysis

**Figure 5:**
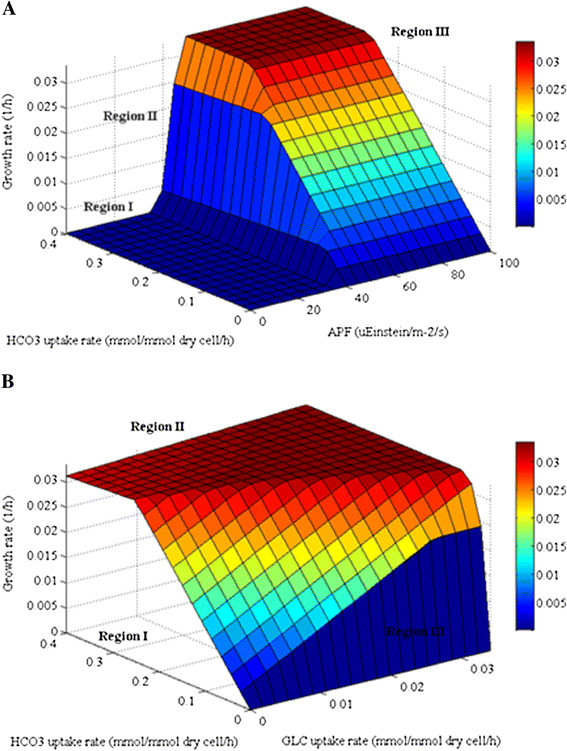
**Phenotypic phase planes for autotrophic and mixotrophic growths of*S. platensis*C1 metabolic network.** (A) autotrophy, the estimated maximal biomass formation rate as a function of light and bicarbonate uptake. (B) mixotrophy, the estimated maximal biomass formation rate as a function of bicarbonate and glucose uptake. HCO3: bicarbonate; GLC: glucose.

Phenotypic phase plane (PhPP) analysis is a useful approach for examining the steady-state solution space when two variables of interest are varied [25]. We employed PhPP to demonstrate the effect of light and bicarbonate ions on the growth of *i*AK692 under autotrophic condition. Within the metabolic network, inorganic carbon was transported from the medium as bicarbonate ions into cells. The intracellular bicarbonate was then dehydrated via carbonic anhydrase enzyme to become CO_2_. The intensity of light input was represented by absorbed photon flux (APF). The surface of a three-dimensional PhPP corresponding to the predicted maximal growth rate as a function of the photon flux (0 – 100 μEinstein/m^2^/s) and the bicarbonate uptake rate (0–0.4 mmol/mmol dry cell/h) was plotted (Fig. 5)A. It was found that the cells exhibited distinct phenotypes depending on the amounts of bicarbonate and light fluxes. It is apparent that the cell growth rate is zero in Region I where the APF is below 40 μEinstein/m^2^/s. It is possible that there is not enough light intensity to generate sufficient amount of ATP required for cell growth in this region. At the APF between 40 and 45 μEinstein/m^2^/s (Region II), the maximal growth was found to be linearly dependent on both the absorbed light and bicarbonate availability. At the APF above 45 μEinstein/m^2^/s (Region III), the cell growth is limited by the bicarbonate ion availability. It is noteworthy to mention that our model fails to describe the photoinhibition at high APFs, although it has been well documented that photoinhibition is observed in autotrophic cultures of *Spirulina*. This is another issue that we will address in a future study.

Furthermore, PhPP for the case of *i*AK692 grown under varying bicarbonate and glucose uptake rates in the presence of constant APF (46.7 μEinstein/m^2^/s) was performed to investigate the relationship between photosynthesis and respiration growth of *i*AK692 during mixotrophy. The PhPP plot shows three distinct regions describing different growth phenotypes (Fig. 5B). In region I where the glucose uptake flux equals zero, *i*AK692 is able to grow using bicarbonate ion as a sole carbon source via photosynthetic pathways. This region can be characterized as autotrophy. In region II, the optimal growth of *i*AK692 was linearly dependent on both bicarbonate and glucose uptake fluxes. However, such dependency does not exist at high uptake rates as the carbon nutrients become saturated. Both photosynthetic and respiratory pathways are active in this region. In region III, we observed that *i*AK692 fails to grow in the absence of bicarbonate ion regardless of the amount of glucose availability. This implies that the respiratory pathways in the *i*AK692 strain could be triggered only if the photosynthetic pathways are active during the mixotrophic growth.

## Conclusions

Herein, we reconstructed a genome-scale metabolic network of the cyanobacterium *S. platensis* C1, which is valuable both scientifically and economically. The network was constructed using i) automatic reconstruction by Pathway Tools software [26,27]; and ii) iterative processes of extensive manual curation based on genomic and bibliome data (Fig. 1). The curated network accounts for 771 metabolic genes, 712 metabolites, and 868 reactions. More than 85 % of the total reactions were associated with genes**.**

We used the COBRA toolbox [13,14] to investigate the global metabolic capability of *S. platensis* C1. This approach allowed an estimation of the flux distribution of the entire metabolism. The simulated results were validated by experimental evidence and showed satisfactory agreement under three different growth conditions; namely, autotrophic, heterotrophic, and mixotrophic. Analyses of the *i*AK692 model enabled us to gain insights into the metabolic phenotypes and essential genes of *S. platensis* C1 grown under these conditions. With a growing *Spirulina* community, the proposed model would not only be useful for studying cellular phenotypes but it could also serve as a platform for “-omics” data integration in order to achieve the beneficial stage of model-driven discovery in *Spirulina* systems biology [8].

## Methods

### Culture conditions and sample analysis

*S. platensis* strain C1 was used in this study. Cells were grown at 35 °C in 1,000 ml Erlenmeyer flasks with a culture volume of 500 ml and continuous stirring. Autotrophic and mixotrophic cultures were grown under fluorescent light at 100 μEinstein/m^2^/s. Zarrouk’s medium [47] was used for autotrophic growth and Zarrouk’s medium with glucose at a final concentration of 20 mM was used for the mixotrophic cultures. For the aerobic-dark cultures (heterotrophic), the flasks were wrapped with aluminum foil and incubated in the dark and sodium bicarbonate in Zarrouk’s medium was substituted by 20 mM of glucose. The control culture was not provided with a carbon source. In this heterotrophic cultivation using various organic or inorganic compounds as a carbon source, the cultures were cultivated in 250 ml flasks containing 100 ml of each tested medium, with three replicates.

The maximum growth rates under autotrophic, heterotrophic, and mixotrophic growth conditions were measured by the optical density at 560 nm and compared with the standard curve. The results from these three conditions are shown in Table 4. For autotrophic growth, the amount of bicarbonate was determined by titration with 0.1 N H_2_SO_4_[48]. The level of phosphate was measured as described elsewhere [49]. The maximum uptake rates of both substrates were calculated as 0.20 and 0.0056 mmol/mmol dry cell/h, respectively, in the exponential phase.

### Metabolic network reconstruction

A metabolic network of *S. platensis* C1 was formulated using a combination of two procedures: automatic and manual reconstruction (see Fig. 1). In order to accelerate the process of metabolic network reconstruction, the annotated data of the draft genome sequence of *S. platensis* C1 (NCBI ID 67617) [24] were used as the input for the Pathway Tools software version 13.0 [26,27], which can automatically generate a preliminary gene-protein-reaction (GPR) association in the network. The PathoLogic algorithm embedded in the software performs the inference process from the entire sequence and functional annotations of *S. platensis* C1 by comparing the data to the MetaCyc database [31] as a key reference. The initial metabolic network consists of connections between the gene sequences, enzymes, metabolites, reactions, and biochemical pathways. Then, the manual reconstruction procedure was performed. Biochemical information related to *Spirulina* from the literature, books and published databases, such as KEGG [32], Brenda [33], CyanoBase [50] and updated Metacyc [31], were used as manually curated data for each pathway, reaction and gene product (enzyme): i) presence/absence pathway and reaction, ii) metabolite and cofactor specificity, iii) directionality of reactions, and iv) GPR association and location. The exchange reactions that allow specific molecules through the system and environment were included in the model according to TransportDB [51]. The Pathway Tools software cannot automatically provide information about protein complexes or isoenzymes. Published information must be used to determine the type of enzyme relationship, which is assumed to be an AND or OR relationship, where the AND relationship indicates cooperation between subunits in protein complexes and the OR relationship indicates the existence of isoenzymes. BLAST [28,29] was used for assigning enzymatic functions of the missing genes by searching nucleotide sequences against NCBI’s database using the expectation value (E-value) of less than e-5 and the similarity and identity score of 50 %. The top best hits were taken to check for the protein domain against pfam database [30] , if it contains the conserved domain, gene are assumed to be functional orthologues. However, the no gene-association reactions were presented in the refined network, although we could not annotate the corresponding gene sequence in the *S. platensis* C1 genome via the homology search because of its physiological evidence. During the manual curation, iterative modeling was performed using FBA for checking the completeness and consistency of the model and experiment. Some reactions were added to fulfill the system connection based on reactions present in closed organisms. The process of curation is iterative until the gaps in a draft metabolic network are filled [52,53]. An accepted metabolic network of *S. platensis* C1 was further used to predict growth behavior under autotrophic, heterotrophic, and mixotrophic conditions. Since no biomass composition data for *S. platensis* C1 are available, the stoichiometry of the biomass formation reaction used in this study was obtained from the work of Cogne *et al*[23].

### Topology analysis

In order to analyze the metabolites connected within the network, we formulated a stoichiometric matrix S, derived from reaction lists of *S. platensis* C1. The column and row represent a reaction and a metabolite, respectively; each element is a stoichiometric coefficient. For each network, metabolite connectivity is defined as the number of metabolites that participate in any given reaction. The number of occurrences of each metabolite was calculated to reveal the highly connected metabolites of the reconstructed network. We also compared the metabolite connectivity pattern between the published genome-scale metabolic networks of *Synechocystis* sp. PCC6803 (*i*Syn669) [20], *E. coli (i*AF1260*)*[36], and yeast (*i*FF708) [37].

### Flux balance analysis (FBA)

Flux balance analysis is a modeling technique that requires a developed stoichiometric metabolic network and a list of constraint parameters of biochemical reactions [11,12]. A set of metabolic reactions are converted into a mathematical stoichiometric format or an S (m × n) matrix, where the rows indicate the metabolites, m, and the columns represent the reactions, n. Based on a pseudo-steady state assumption, the change in growth rate is much smaller than the change in metabolite concentration and flux. Thus, the model could be written as S x v = 0, where v corresponds to a vector of all reaction fluxes in the network. Since the metabolic networks usually possess higher the number of independent reactions than the number of metabolites, the rank of a developed stoichiometric matrix is thus less than the number of reactions fluxes, giving rise to an underdetermined system. Using linear programming, the flux vector can be found by specifying an objective function (*z*) that can be minimized or maximized. In the case of minimization, the linear equation can be written as min *z* = c^T^v , where c is a row vector representing the influence of individual fluxes on the objective function. The metabolic flux distributions of the network are estimated under given conditions. In addition, the constraint parameters indicate the allowable range of flux values and are needed for convex solution space. The constraints for the upper and lower boundaries of reversible and irreversible reactions were defined as -∞ ≤ v_i_ ≤ ∞ and 0 ≤ v_i_ ≤ ∞, respectively. More details on the FBA approach have been described elsewhere [54,55].

### Minimization of metabolic adjustment (MOMA)

The algorithm of minimization of metabolic adjustment was introduced by Segre *et al*[46]. This approach uses quadratic programming to search for a point in the feasible solution space of the mutant, which is nearest to an optimal point in the wild-type feasible solution space. The minimal distance is evaluated from the closest point and defined as the Euclidian distance. MOMA is also based on the same stoichiometric constraints as FBA, but it relaxes the assumption of optimal growth flux for the mutants. This method displays a suboptimal flux distribution that is intermediate between wild-type optimum and mutant optimum. In this study, we used the MOMA algorithm available in the COBRA toolbox [13,14] to carry out the gene essentiality analysis.

### Model simulation

In this work, we used the COBRA toolbox [13,14] with the Systems Biology Markup Language (SBML) Toolbox v2 [56] on MATLAB to automatically construct the stoichiometric matrix of the reconstructed metabolic network. This tool uses FBA, which uses glpk (http://www.gnu.org/software/glpk/) as the linear programming solver to estimate the optimal flux distributions under the maximized biomass objective. Simulations of three different growth conditions: heterotrophic (with glucose, aerobically in the dark), autotrophic (without glucose in the light) and mixotrophic (with glucose in the light) were performed according to the minimal growth-dependent medium. The uptake rates of carbon, nitrogen, phosphate, and sulfate sources were set and varied under each condition as shown in Table 4. The minimal medium of heterotrophic and mixotrophic growth was supplemented with glucose as the other carbon source. The light reaction was lumped and the photon flux was constrained between zero and 100 μEinstein/m^2^/s for autotrophic and mixotrophic conditions, while it was set to zero for heterotrophic cell growth. The other external metabolites involved in transport reactions such as H_2_O, Na^+^, Mg^2+^, H^+^, Zn^2+^, and Fe^2+^ (see Addition file 1), except for the substrates, were allowed to freely enter or leave the system. The uptake rates of all metabolites absent in the medium were set to zero. The flux values were expressed in mmol/mmol dry cell/h. All the three models in SBML format are provided in Additional files 5-7.

### Active reaction determination

In order to investigate the system-level change in response to the growth conditions we identified modes of metabolic operation under autotrophy and mixotrophy in terms of active reaction presented the non-zero flux value of the simulations. The different *in silico* constraints of the simulation were set according to the carbon source utilized, as described above.

### Flux variability analysis (FVA)

Flux variability analysis is used to examine the full range of numerical values for each reaction flux in the metabolic system while still satisfying the given constraints set and optimizing for a particular objective [44]. Determining the range of flux values (v) through each reaction, maximum value of the biomass objective function (*z*) is first computed and is used as s further constraint with multiple optimizations to minimize and maximize flux values of every reaction through FBA. The mathematical equation can be written as max(*z*^T^v). The difference between the calculated minimum and maximum values for each flux defines the flux variability of that reaction. In this study, we used the COBRA toolbox [13,14] to perform FVA.

### Gene essentiality analysis

The effect of genetic changes such as gene deletion can be simulated by constraining reactions associated to gene of interest to be zero [45]. To determine the effect of genetic perturbation, all reactions associated with each gene in the *i*AK692 model were individually removed while still optimizing the growth rate. This *in silico* analysis was performed using the MOMA algorithm running through the COBRA toolbox [13,14] under autotrophic and mixotrophic conditions. An essential gene (lethal gene) was defined if no positive flux value for biomass formation could be obtained for a given mutant.

### Phenotypic phase plane (PhPP)

A robustness analysis was performed to determine the sensitivity of the predicted growth in changing the fluxes through a pre-defined range. Phenotypic phase plane is the method which has been used to obtain the sensitivity analysis as a function of dual variables [25]. PhPP analysis was done by varying two particular reactions of interest and iteratively calculating the objective function. The shadow prices of the dual fluxes were evaluated for each solution. In metabolic network, a shadow prices is the rate at which the objective function (*z*) changes in response to an increase availability of each metabolite. Mathematically equation used to calculate the shadow price can be written as: γ_i_ = d*z*/db_i_ where γ_i_ is the *i*th shadow price and b_i_ is the *i*th metabolite of the metabolic network. In this study, we performed PhPP using the COBRA toolbox [13,14]. The simulation was carried out with autotrophy by setting the boundaries of the absorbed photon fluxes between zero to 100 μEinstein/m^2^/s; and of the bicarbonate uptake rates between zero to 0.4 mmol/mmol dry cell/h. For mixotrophy, PhPP analysis was performed by varying the boundaries of the glucose uptake rates between zero to 0.034 mmol/mmol dry cell/h and the bicarbonate uptake rates between zero to 0.4 mmol/ mmol dry cell/h.

## **Competing interests**

The authors declare that they have no competing interests.

## Authors’ contributions

AK performed the reconstruction of the genome-scale metabolic model and the network analysis and wrote the manuscript; AK, AP, and AM designed the study and were involved in model simulation and evaluation. AK, AP, and CK performed the *Spirulina* cell cultivation experiments. All authors read and approved the final manuscript.

## Supplementary Material

Additional file 1 Additional file 1***i*AK692 model.** Excel file containing a list of the biochemical reactions in the final version of the metabolic network reconstruction, *i*AK692. Click here for file

Additional file 2 **Additional file 2: Fluxome distribution under three different growth simulations.** This Excel file contains a list of all flux distribution (in mmol/mmol dry cell/h) profiles under the autotrophic, heterotrophic, and mixotrophic culture conditions. Click here for file

Additional file 3 **Additional file 3: Flux variability analysis.** Excel file contains flux variability of the entire metabolic network for autotrophic and mixotrophic growths. Click here for file

Additional file 4 **Additional file 4: Gene deletion analysis using MOMA approach in*S.platensis*C1 metabolic model.** Excel file contains the grRatio, grRateKO and grRateWT values for *S. platensis* C1 single gene deletion during autotrophic and mixotrophic growths. Click here for file

Additional file 5 **Additional file 5: SBML Model for the autotrophic simulation.**This file contains the stoichiometric iAK692 model in the SBML format with all of the constraints needed for simulation using the COBRA toolbox. Click here for file

Additional file 6 **Additional file 6: SBML Model for the autotrophic simulation.**This file contains the stoichiometric iAK692 model in the SBML format with all of the constraints needed for simulation using the COBRA toolbox. Click here for file

Additional file 7 **Additional file 7: SBML Model for the autotrophic simulation.**This file contains the stoichiometric iAK692 model in the SBML format with all of the constraints needed for simulation using the COBRA toolbox. Click here for file

## References

[B1] Pinero Estrada JE Bermejo Bescos P del Fresno AM Villar Antioxidant activity of different fractions of Spirulina platensis protean extract Farmaco 2001 56 497 500 10.1016/S0014-827X(01)01084-9 11482785

[B2] Tietze HW Spirulina-Micro Food Macro Blessing 2004 4 Harald W. Tietz Publishing, Australia

[B3] Habib MAB Parvin M Huntington TC Hasan MR A review on culture, production and use of spirulina as food for humans and feeds for domestic animals and fish 2008 Food and agriculture organization of the united nations, Retrieved November 20, 2011

[B4] Kulshreshtha A Zacharia AJ Jarouliya U Bhadauriya P Prasad GB Bisen PS Spirulina in health care management Current pharmaceutical biotechnology 2008 9 400 405 10.2174/138920108785915111 18855693

[B5] Ghirardi ML Zhang L Lee JW Flynn T Seibert M Greenbaum E Melis A Microalgae: a green source of renewable H(2) Trends Biotechnol 2000 18 506 511 10.1016/S0167-7799(00)01511-0 11102662

[B6] Santillan C Mass production of Spirulina Experientia 1982 38 40 43 10.1007/BF01944524

[B7] Feist AM Herrgard MJ Thiele I Reed JL Palsson BO Reconstruction of biochemical networks in microorganisms Nat Rev Microbiol 2009 7 129 143 19116616 10.1038/nrmicro1949 PMC3119670

[B8] Reed JL Famili I Thiele I Palsson BO Towards multidimensional genome annotation Nat Rev Genet 2006 7 130 141 10.1038/nrg1769 16418748

[B9] Patil KR Akesson M Nielsen J Use of genome-scale microbial models for metabolic engineering Curr Opin Biotechnol 2004 15 64 69 10.1016/j.copbio.2003.11.003 15102469

[B10] Palsson BO System Biology: Determining the Capabilities of Reconstructed Networks 2006 Cambrige University Press, London/New York

[B11] Kim HU Kim TY Lee SY Metabolic flux analysis and metabolic engineering of microorganism Molecular BioSystems 2008 4 113 120 10.1039/b712395g 18213404

[B12] Price ND Reed JL Palsson BO Genome-scale models of microbial cells: evaluating the consequences of constraints Nat Rev Microbiol 2004 2 886 897 10.1038/nrmicro1023 15494745

[B13] Becker SA Feist AM Mo ML Hannum G Palsson BO Herrgard MJ Quantitative prediction of cellular metabolism with constraint-based models: the COBRA Toolbox Nat Protoc 2007 2 727 738 10.1038/nprot.2007.99 17406635

[B14] Schellenberger J Que R Fleming RM Thiele I Orth JD Feist AM Zielinski DC Bordbar A Lewis NE Rahmanian S Quantitative prediction of cellular metabolism with constraint-based models: the COBRA Toolbox v2.0 Nat Protoc 2011 6 1290 1307 10.1038/nprot.2011.308 21886097 PMC3319681

[B15] Oberhardt MA Palsson BO Papin JA Applications of genome-scale metabolic reconstructions Molecular systems biology 2009 5 320 19888215 10.1038/msb.2009.77 PMC2795471

[B16] Durot M Bourguignon PY Schachter V Genome-scale models of bacterial metabolism: reconstruction and applications FEMS microbiology reviews 2009 33 164 190 10.1111/j.1574-6976.2008.00146.x 19067749 PMC2704943

[B17] Osterlund T Nookaew I Nielsen J Fifteen years of large scale metabolic modeling of yeast: Developments and impacts 2011 Biotechnology advances, 10.1016/j.biotechadv.2011.07.021 21846501

[B18] Kim TY Sohn SB Kim YB Kim WJ 2011 Recent advances in reconstruction and applications of genome-scale metabolic models. Curr Opin Biotechnol, Lee SY 10.1016/j.copbio.2011.10.007 22054827

[B19] Fu P Genome-scale modeling of Synechocystis sp. PCC 6803 and prediction of pathway insertion Journal of Chemical Technology and Biotechnology 2008 84 411 473 483

[B20] Montagud A Navarro E Fernandez de Cordoba P Urchueguia JF Patil KR Reconstruction and analysis of genome-scale metabolic model of a photosynthetic bacterium BMC systems biology 2010 4 156 10.1186/1752-0509-4-156 21083885 PMC3009638

[B21] Yoshikawa K Kojima Y Nakajima T Furusawa C Hirasawa T Shimizu H Reconstruction and verification of a genome-scale metabolic model for Synechocystis sp. PCC6803 Applied microbiology and biotechnology 2011 92 347 358 10.1007/s00253-011-3559-x 21881889

[B22] Meechai A Pongakarakun S Deshnium P Cheevadhanarak S Bhumiratana S Metabolic flux distribution for gamma-linolenic acid synthetic pathways in Spirulina Platensis Biotechnology and Bioprocess Engineering 2004 9 506 513 10.1007/BF02933494

[B23] Cogne G Gros JB Dussap CG Identification of a metabolic network structure representative of Arthrospira (spirulina) platensis metabolism Biotechnol Bioeng 2003 84 667 676 10.1002/bit.10808 14595779

[B24] Cheevadhanarak S Paithoonrangsarid K Prommeenate P Kaewngam W Musigkain A Tragoonrung S Tabata S Kaneko T Chaijaruwanich J Sangsrakru D Draft genome sequence of Arthrospira platensis C1 (PCC9438) 2012 Standards in Genomic Sciences, North America 6 10.4056/sigs.2525955 PMC3368399 22675597

[B25] Edwards JS Ramakrishna R Palsson BO Characterizing the metabolic phenotype: a phenotype phase plane analysis Biotechnol Bioeng 2002 77 27 36 10.1002/bit.10047 11745171

[B26] Karp PD Paley S Romero P The Pathway Tools software Bioinformatics 2002 18 Suppl 1 S225 232 10.1093/bioinformatics/18.suppl_1.S225 12169551

[B27] Paley SM Karp PD The Pathway Tools cellular overview diagram and Omics Viewer Nucleic Acids Res 2006 34 3771 3778 10.1093/nar/gkl334 16893960 PMC1557788

[B28] Altschul SF Madden TL Schaffer AA Zhang J Zhang Z Miller W Lipman DJ Gapped BLAST and PSI-BLAST: a new generation of protein database search programs Nucleic Acids Res 1997 25 3389 3402 10.1093/nar/25.17.3389 9254694 PMC146917

[B29] Mount DW Using the Basic Local Alignment Search Tool (BLAST) 2007 CSH protocols, pdb top17 10.1101/pdb.top17 21357135

[B30] Finn RD Mistry J Tate J Coggill P Heger A Pollington JE Gavin OL Gunasekaran P Ceric G Forslund K The Pfam protein families database Nucleic Acids Res 2010 38 D211 222 10.1093/nar/gkp985 19920124 PMC2808889

[B31] Caspi R Foerster H Fulcher CA Kaipa P Krummenacker M Latendresse M Paley S Rhee SY Shearer AG Tissier C The MetaCyc Database of metabolic pathways and enzymes and the BioCyc collection of Pathway/Genome Databases Nucleic Acids Res 2008 36 D623 631 17965431 10.1093/nar/gkm900 PMC2238876

[B32] Kanehisa M Goto S Hattori M Aoki-Kinoshita KF Itoh M Kawashima S Katayama T Araki M Hirakawa M From genomics to chemical genomics: new developments in KEGG Nucleic Acids Res 2006 34 D354 357 10.1093/nar/gkj102 16381885 PMC1347464

[B33] Chang A Scheer M Grote A Schomburg I Schomburg D BRENDA, AMENDA and FRENDA the enzyme information system: new content and tools in 2009 Nucleic Acids Res 2009 37 D588 592 10.1093/nar/gkn820 18984617 PMC2686525

[B34] Yang C Hua Q Shimizu K Energetics and Carbon Metabolism During Growth of Microalgal Cells under Photoautotrophic, Mixotrophic and Cyclic Light-Autotrophic/Dark-Heterotrophic Condition Biochemical Engineering Journal 2000 6 87 102 10.1016/S1369-703X(00)00080-2 10959082

[B35] Enzyme Nomenclature 1992: IUB Recommendations of the Nomenclature Committee of the International Union of Biochemistry and Molecular Biology on the Nomenclature and Classification of Enzymes 1992 Academic, San Diego

[B36] Feist AM Henry CS Reed JL Krummenacker M Joyce AR Karp PD Broadbelt LJ Hatzimanikatis V Palsson BO A genome-scale metabolic reconstruction for Escherichia coli K-12 MG1655 that accounts for 1260 ORFs and thermodynamic information Molecular systems biology 2007 3 121 17593909 10.1038/msb4100155 PMC1911197

[B37] Forster J Famili I Fu P Palsson BO Nielsen J Genome-scale reconstruction of the Saccharomyces cerevisiae metabolic network Genome research 2003 13 244 253 10.1101/gr.234503 12566402 PMC420374

[B38] Chojnacka K Noworyta A Evaluation of Spirulina sp. growth in photoautotrophic, heterotrophic and mixotrophic cultures Enzyme and Microbial Technology 2004 34 461 465 10.1016/j.enzmictec.2003.12.002

[B39] Lodi A Binaghi L Faveri DD Carvalho JCM Converti A Fed-Batch Mixotrophic Cultivation ofArthrospira (Spirulina) Platensis(Cyanophycea) with Carbon Source Pulse Feeding Annals of Microbiology 2005 55 3 181 185

[B40] Muhling M Belay A Whitton BA ScreeningArtrospira(Spirulina) strains for Heterotrophy Journal of Applied Phycology 2005 17 129 135 10.1007/s10811-005-7214-8

[B41] Marquez FJ Sasaki K Kakizono T Nishio N Nagai S Growth characteristics of Spirulina platensis in mixotrophic and heterotrophic conditions Journal of Fermentation and Bioengineering 1993 76 408 410 10.1016/0922-338X(93)90034-6

[B42] Milne CB Eddy JA Raju R Ardekani S Kim PJ Senger RS Jin YS Blaschek HP Price ND Metabolic network reconstruction and genome-scale model of butanol-producing strain Clostridium beijerinckii NCIMB 8052 BMC systems biology 2011 5 130 10.1186/1752-0509-5-130 21846360 PMC3212993

[B43] Nishikawa T Gulbahce N Motter AE Spontaneous reaction silencing in metabolic optimization PLoS computational biology 2008 4 e1000236 10.1371/journal.pcbi.1000236 19057639 PMC2582435

[B44] Mahadevan R Schilling CH The effects of alternate optimal solutions in constraint-based genome-scale metabolic models Metab Eng 2003 5 264 276 10.1016/j.ymben.2003.09.002 14642354

[B45] Joyce AR Reed JL White A Edwards R Osterman A Baba T Mori H Lesely SA Palsson BO Agarwalla S Experimental and computational assessment of conditionally essential genes in Escherichia coli Journal of bacteriology 2006 188 8259 8271 10.1128/JB.00740-06 17012394 PMC1698209

[B46] Segre D Vitkup D Church GM Analysis of optimality in natural and perturbed metabolic networks Proceedings of the National Academy of Sciences of the United States of America 2002 99 15112 15117 10.1073/pnas.232349399 12415116 PMC137552

[B47] Vonshak A Laboratory Techniques for the Cultivation of Microalgae: CRC Handbook of Microalgae Mass Cultures 1986 CRC Press, Florida, USA

[B48] Michel DuBois Gilles KA Hamilton JK Rebers PA Smith F Colorimetric Method for Determination of Sugars and Related Substances Analytical Chemistry 1956 28 350 356 10.1021/ac60111a017

[B49] Franson M Standard Methods for the Examination of Water and Wastewater, 20thedn.American Public Heath Association, American Water Works Association, Water Enviroment Federation 1998 United Book Press, Baltimore, Maryland, USA

[B50] Nakamura Y Kaneko T Hirosawa M Miyajima N Tabata S CyanoBase, a www database containing the complete nucleotide sequence of the genome of Synechocystis sp. strain PCC6803 Nucleic Acids Res 1998 26 63 67 10.1093/nar/26.1.63 9399802 PMC147195

[B51] Ren Q Chen K Paulsen IT TransportDB: a comprehensive database resource for cytoplasmic membrane transport systems and outer membrane channels Nucleic Acids Res 2007 35 D274 279 10.1093/nar/gkl925 17135193 PMC1747178

[B52] Feist AM Scholten JC Palsson BO Brockman FJ Ideker T Modeling methanogenesis with a genome-scale metabolic reconstruction of Methanosarcina barkeri Molecular systems biology 2006 2 2006 0004 16738551 10.1038/msb4100046 PMC1681478

[B53] Duarte NC Becker SA Jamshidi N Thiele I Mo ML Vo TD Srivas R Palsson BO Global reconstruction of the human metabolic network based on genomic and bibliomic data Proceedings of the National Academy of Sciences of the United States of America 2007 104 1777 1782 10.1073/pnas.0610772104 17267599 PMC1794290

[B54] Kauffman KJ Prakash P Edwards JS Advances in flux balance analysis Curr Opin Biotechnol 2003 14 491 496 10.1016/j.copbio.2003.08.001 14580578

[B55] Stephanopoulos GN Aristidou AA Nielsen J Metabolic Engineering Principles and Methodologies 1998 Acadimic press, San Diego

[B56] Keating SM Bornstein BJ Finney A Hucka M SBMLToolbox: an SBML toolbox for MATLAB users Bioinformatics 2006 22 1275 1277 10.1093/bioinformatics/btl111 16574696

